# Observation of retinal neovascularization using optical coherence tomography angiography after panretinal photocoagulation for proliferative diabetic retinopathy

**DOI:** 10.1186/s12886-021-01964-w

**Published:** 2021-06-07

**Authors:** H. E. Feng, Y. U. Weihong, Fangtian Dong

**Affiliations:** Department of Ophthalmology, State Key Laboratory of Complex Severe and Rare Diseases, Peking Union Medical College Hospital, Key Laboratory of Ocular Fundus Diseases, Chinese Academy of Medical Sciences, Beijing, 100730 China

**Keywords:** Optical coherence tomography angiography, Panretinal photocoagulation, Neovascularization

## Abstract

**Background:**

To describe the longitudinal changes in retinal neovascularization elsewhere (NVE) as observed on optical coherence tomography angiography (OCTA) in proliferative diabetic retinopathy (PDR) treated by panretinal photocoagulation (PRP).

**Methods:**

Each patient included in this prospective clinical study was newly diagnosed with PDR and NVE confirmed by both fundus fluorescein angiography (FFA) and OCTA. They received four sessions of PRP using a multiwavelength laser. Best-corrected visual acuity (BCVA) and OCTA images of the NVE were obtained before each PRP session and at 1 month, 3 months, and 6 months after the PRP treatment. Generalized estimating equations (GEE) was used to investigate the differences between the BCVA and NVE areas before and after PRP.

**Results:**

Thirty-two eyes of 32 patients with a mean age of 50.56 ± 7.05 years were included. We found a statistically significant reduction in the NVE area at all time points compared with the baseline except at 6 months (all *P* < 0.05). Further analysis demonstrated no statistically significant change in the NVE area between two adjacent timepoints except from baseline to post-1st PRP (*P* < 0.05). BCVA at 3 months showed a statistically significant improvement compared with baseline (*P* < 0.05), but no significant changes in BCVA were observed during the other visits.

**Conclusions:**

We found an overall regression in the NVE area following PRP starting as early as 1 week after the 1st session and lasting up to 3 months. OCTA provides quantitative information on vascular changes and could be a practical method for the longitudinal evaluation of neovascularization.

## Background

Diabetic retinopathy (DR) is the leading cause of visual impairment and blindness in the working population [1]. DR is responsible for vision loss in 12.6% of diabetic patients and it tends to occur in young patients in Asian countries [1]. Proliferative diabetic retinopathy (PDR), characterized by retinal neovascularization at the disc (NVD) or elsewhere in the retina (NVE), is the most common form [2]. Panretinal photocoagulation (PRP) has been the standard treatment for PDR during the past several decades. Although anti-VEGF agents have demonstrated a positive effect in regressing retinal neovascularization in recent years, PRP is still recommended by 98% of retina specialists as the primary management of PDR [3]. In China, treatment with repeated injections of anti-VEGF agents is impractical in many patients because they are not covered by medical insurance, but the cost-effectiveness and relatively long duration of efficacy make PRP a sound and sensible choice for these patients.

The goal of PRP is to modify the natural history of PDR by regressing the neovascularization (NV) and destroying areas of the peripheral retina to reduce the triggers for NV formation [4]. According to the ETDRS, 60% of PDR patients responded to PRP with regression of NV within 3 months [4], but the temporal profile of NV changes has not been addressed in a detailed fashion. The introduction of optical coherence tomography angiography (OCTA) has provided clinicians with a noninvasive tool to study the morphological details of retinal neovascular complex [5]. Quantitative information on NV can be repeatedly obtained with OCTA without concern about the intravenous dye-related adverse outcomes that are related to fundus fluorescein angiography (FFA). Furthermore, the absence of leakage in OCTA facilitates a precise delineation of the NV area, which enables the frequent and repeated evaluation of NV changes before and after PRP sessions.

In the present study, we sought to explore the short-term and long-term effects of PRP in regressing retinal NV, and OCTA was performed to quantify and monitor the NVE changes over time in response to the PRP treatment.

## Methods

This was a prospective analysis of a case series including the patients diagnosed with PDR in the Department of Ophthalmology at Peking Union Medical College Hospital between December 2017 and February 2019. The study was approved by the Ethics Committee of Peking Union Medical College Hospital. All procedures were carried out in accordance with the tenets of the Declaration of Helsinki. Informed consent was obtained from all participants.

The inclusion criteria included treatment-naïve patients with newly diagnosed PDR and NVE, and those who were confirmed by both FFA and OCTA. To reduce bias, each patient chose one eye for observation during this study. The exclusion criteria were as follows: NVE outside the OCTA scan range; NVD (retinal neovascularization at the disc); other causes of NVE, such as retinal vein occlusion; fibrovascular proliferation with retinal traction; fibrosis, scarring, atrophy and hard exudates with central involvement; diabetic macular oedema (DME) involving the central macula; and a previous history of optic neuropathy or uncontrolled glaucoma.

The eligible patients received PRP in 4 sessions with an interval of 1 week between sessions. PRP was performed in the order of the nasal, inferior, superior, and temporal sides with 300 ~ 400 scatter laser burns per session by a retina specialist (Feng H.) using a VISULAS® Trion multiwavelength laser (Carl Zeiss Meditec Inc., Dublin, CA) and each treatment was completed with a total of 1200 ~ 1600 spots. During the treatment, laser parameters were individually adjusted to obtain mild white spots with a 300-μm spot size separated by 300 μm.

Patients underwent comprehensive ophthalmic examinations that included best-corrected visual acuity (BCVA) (Snellen visual acuity ratios), intraocular pressure, slit-lamp biomicroscopy, indirect ophthalmoscopy and an OCTA examination. Data were collected at 7 timepoints: at baseline before they underwent the first PRP session during the same visit; at each visit before the second, third, and fourth PRP sessions; and then at 1 month, 3 months, and 6 months after the last PRP session. The primary outcome was NVE changes from baseline to 6 months, and the secondary outcome was BCVA changes over time.

OCTA images were acquired by a retina specialist (Feng H.) using the RTVue-XR Avanti spectral-domain OCT with AngioVue software 2.0 (Optovue Inc., Fremont, California, USA). The “HD Angio Retina 6 × 6 mm” mode was used to encompass the NVE area also visible on the corresponding FFA. During the image acquisition process, the scanning position of the OCTA was manually adjusted to obtain high-quality NVE images. Images with low scan quality (SSI less than 40) or significant motion artefacts were discarded, and the best images for the detection of new vessel structures were selected for analysis. If there were multiple NVEs, only one NVE with the highest baseline image quality was selected for observation.

**Fig. 1:**
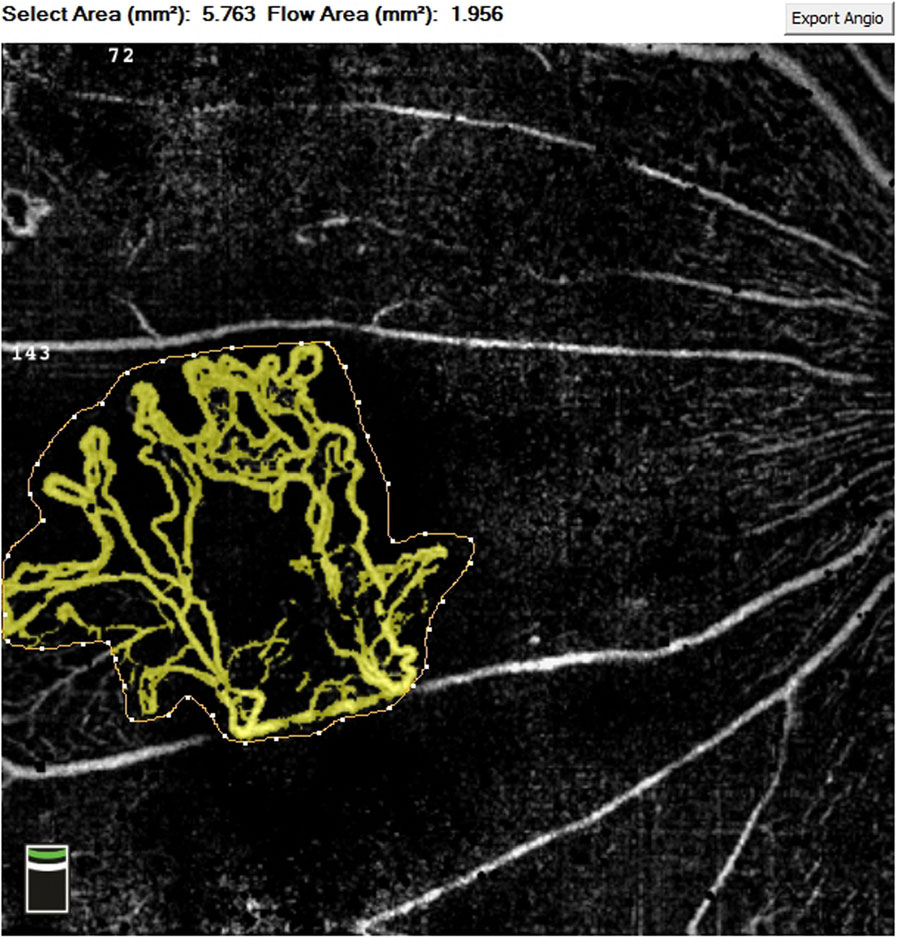
Measurement of the neovascularization area on OCTA. A select area of 0.998 mm^2^ (highlighted) was manually outlined by the retina specialist, and the flow area of 0.653 mm^2^ was automatically calculated by Angiovue software

To ensure the validity and repeatability of the results, the images were analysed independently by two independent retina specialists who were masked to the clinical status of the patients (Weihong Y. and Fangtian D.). B-scan OCT images with overlaying flow signals were used to confirm the presence of new vessels, which were seen as structures with positive flow signals existing on the surface of the retina or protruding into the vitreous cavity. Segmentation of the inner boundary of the B-scan was manually moved in the vitreous cavity above the new vessels, and the outer boundary was manually adjusted just below the internal limiting membrane to minimize the depiction of the superficial vascular plexus [6]. A region of interest was manually outlined by the retina specialists to encompass the new vessels, and then the flow area was automatically calculated by multiplying the number of pixels for which the decorrelation value was above that of the background wih Angiovue software (Fig. 1). Measurements of the NVE area represent the average value obtained by the two observers. Before statistical analysis, the images obtained by OCTA were corrected according to the Littman formula and Sampson DM et al.’s conclusion [7, 8].

We used generalized estimating equations (GEE) to take into account the correlation of changes over time by assuming an autoregressive (1) correlation structure of the responses from the subjects. Multiple comparisions were adjusted by uisng Tukey’s method. Intraclass correlation coefficients (ICC) were calculated to assess the agreement between the observers. A *P* value of less than 0.05 was considered statistically significant.

## Results

OCTA images from 6 patients were discarded because of low scan quality or significant motion artefacts, and a total of 32 patients (32 eyes) with a mean age of 50.56 ± 7.05 years were enrolled in this study. At baseline, 12 eyes were high-risk PDR. None of the patients had vitreous haemorrhages or other complications during the treatment. The mean baseline NVE area was 1.448 ± 1.101 mm^2^, and the mean baseline BCVA on logMAR was 0.286 ± 0.164. None of the patients underwent additional PRP or other treatment procedures during the follow-up period.

**Fig. 2:**
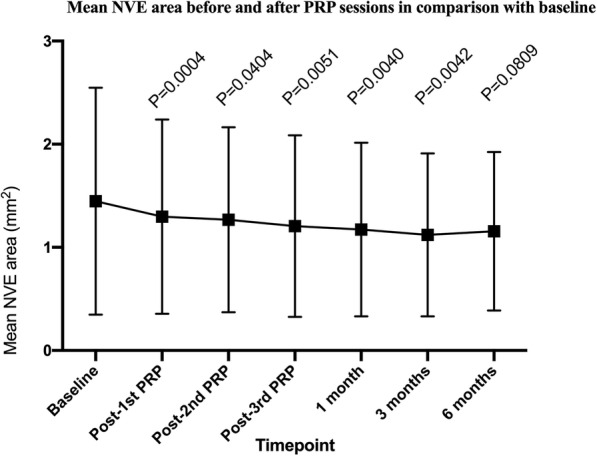
Mean NVE areas (mm^2^) before and after PRP sessions in comparison with baseline

There was excellent agreement between the two observers with respect to the NVE area. The mean NVE area before and after the PRP sessions along with the corresponding intraclass correlation coefficients (ICC) between observers are summarized in Table 1. Compared with baseline, a statistically significant reduction in NVE size was observed at all time points (*P* < 0.05), excluding that at 6 months (Fig. 2). On further analysis, the change in the NVE area was statistically significant from baseline to post-1st PRP (*P* = 0.0004; Table 1). There was no statistically significant difference in NVE area between other adjacent timepoints. BCVA at 3 months showed a statistically significant improvement compared with baseline (*P* < 0.05), but no significant changes in BCVA were observed during the other visits.

**Table 1:** The difference in the mean NVE area in comparison with the previous time point

	Mean NVE area	ICC between observers	*P*-value
Baseline	1.448 ± 1.101	0.927	–
Post-1st PRP	1.297 ± 0.942	0.912	0.0004
Post-2nd PRP	1.268 ± 0.897	0.938	0.9906
Post-3rd PRP	1.206 ± 0.880	0.918	0.4447
1 months	1.172 ± 0.842	0.963	0.9685
3 months	1.121 ± 0.790	0.921	0.3384
6 months	1.155 ± 0.769	0.931	0.7951

## Discussion

In this study, we used OCTA to study the evolution of neovascularization in PDR after PRP therapy throughout a 6-month follow-up period. It was demonstrated that the NVE regression started as early as 1 week following the first PRP session and lasted for at least 3 months.

PRP was recommended in the latest Diabetic Retinopathy Clinical Guidelines released by the American Academy of Ophthalmology in 2017 [9] as the primary treatment for PDR. However, this destructive treatment may be associated with side effects such as pain, transient blurring, macular oedema and loss of peripheral or night vision. Although it has been recommended by ETDRS to repeat PRP sessions from 3 months on for PDR patients, treatment regimens, including the interval between sessions and the timing of additional laser, varied across different studies. In this context, precise quantification of the retinal NV area may be helpful for evaluating the efficacy of different treatment regimens.

Several authors have used FFA to investigate NV changes at 1 ~ 12 months after PRP, but the invasive nature and dye leakage related to FFA hindered frequent and accurate measurement of NV area [10–12]. With the recent availability of OCTA, retinal NV could be regularly followed up at short intervals. Russell JF et al. imaged the NV in PDR patients from 1 week to 3 months after PRP with both FFA and OCTA, supporting the use of OCTA for longitudinal evaluation of NV [13]. In the current study, the NVE area was serially measured at 7 time points, and to our knowledge, this is the first study that has used OCTA to describe in detail the evolution of retinal NV during and after PRP sessions.

Our findings suggest an early and relatively durable response of retinal NV to PRP therapy in treatment-naïve PDR patients; that is, a significant regression of NVE can be detected as early as 1 week after the first PRP session and it lasts up to 3 months. Significant changes in NVE occur from baseline to post-1st PRP. Therefore, it is possible to determine whether a retinal NV is responsive to PRP therapy as early as 1 week after the first session. In addition, when considering whether and when to re-treat the NV an additional session because the therapeutic response is starting to wear off, our study results indicate the re-treatment interval should be at least 3 months, which is consistent with the recommendations by ETDRS. If local intensive laser treatment for NVE was given as suggested by ETDRS, a more significant NVE regression could be anticipated.

Unlike the significant regression of NVE, BCVA in our patients showed no evident improvement except at 3 months [4], which may be due to the inevitable side effects related to laser treatment. However, BCVA in these patients was not significantly worsening during the PRP sessions, and the BCVA at 6 months was still comparable with baseline, suggesting that the laser regimen in this study was reasonable and effective in regressing NV and avoiding side effects as well.

Previous studies of FFA evaluating the response of retinal NV to PRP observed that the anti-NV effect of PRP could last no more than 6 months [10–12], which is in line with the current study and suggests a relatively durable response of retinal NV to PRP treatment. As OCTA is a new imaging modality, it has been used by only a few authors to observe NV changes after PRP treatment. Russell JF et al. imaged the NV in 20 eyes from 1 week to 3 months after PRP using both FFA and OCTA and reported similar progression or regression of NV with both methods [13]. Fawzi AA et al. found that PRP increased macular blood flow which could be measured by several OCTA parameters [14]. Our study first evaluated the NV changes during PRP sessions and demonstrated a very quick response of NV to PRP in treatment-naïve patients, supporting the use of PRP as primary treatment in such patients. Moreover, the comparison between two adjacent timepoints further demonstrated the short-term and long-term effects of PRP. The laser was administered on the nasal side during the 1st PRP session, which might cover more NV around the optic disc and could be the reason why more significant NVE regression was observed after this session. In contrast, the increase in the NVE area between 3 months and 6 months suggested that additional laser treatment should be considered during this period.

OCTA was used in this study to establish the changes in NVE over time following PRP treatment. We found that the NVE area could be rapidly quantified on OCTA, and it was feasible to follow up patients at short intervals without concern about the potential FFA-related adverse events. Therefore, OCTA could be a useful imaging modality for monitoring the efficacy of treatment regimens in PDR patients, which reinforces the results obtained by Russel JF et al. [13]. Since it was reported that 60% of patients with PDR responded to PRP treatment with NV regression within 3 months [12], OCTA could be potentially used in the non-responsive cases to monitor the changes in NV for the reference of treatment regimens, such as the timing of additional laser treatments and combinations of other treatments.

**Fig. 3:**
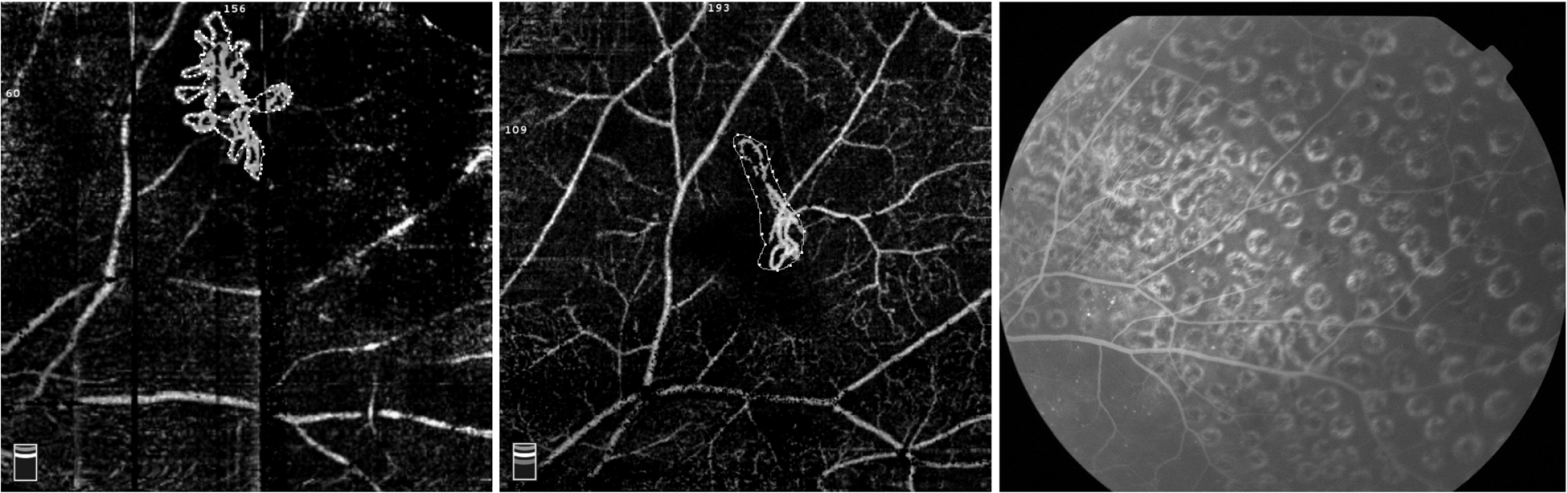
Neovascularization at baseline OCTA with a select area of 0.998 mm^2^ (highlighted) and a flow area of 0.653 mm^2^ (left); OCTA still showed an abnormal vascular shape at an interval of 1 year after PRP, with a select area of 0.675 mm^2^ (highlighted) and a flow area of 0.272 mm^2^ (middle), and no evidence of fluorescein leakage was seen on the FFA (right)

This pilot study has several limitations. First, the small sample size and relatively short follow-up period did not allow for a comprehensive evaluation of NVE changes following PRP treatment. Additional studies to analyse larger datasets are needed to reinforce our results. A second limitation is that the observation of NVE was restricted within a limited field of view and the peripheral NVE could not be visualized. Fortunately, OCTA’s latest technologies, such as extended field imaging (EFI), have solved this problem to a certain extent [15, 16]. Third, OCTA images may also affected by several types of artefacts. Although images with severe artefacts affecting the measurement have been excluded, the potential effect of some artefacts should be carefully evaluated. Some authors have adjusted the image size according to individual axial length [8]. Axial length correction was not performed in this study because all included cases had a refractive error less than ±3.0 D, but this might have an impact on the area calculation of the new vessel area. The NVE area in this study was automatically calculated using the Angiovue software by multiplying the number of pixels for which the decorrelation value was above that of the background. This was dependent on the image quality and might influence the repeatability of measurements in some cases. The leakage of new vessels could not be identified with OCTA as it can with FFA; thus, the activity of the NV could not be directly evaluated, and the presence of residual vessels on OCTA may or may not correlate with activity or risk of vision loss. Therefore, in cases with persistent NV on OCTA, FFA is warranted to assess its activity, and the prognosis of such blood vessels needs further investigation. In one of our cases, a shrinking NVE was present on OCTA images of 1 year after PRP, but no fluorescein leakage was observed on FFA at the same time (Fig. 3), suggesting that the endothelial cells of these residual blood vessels may have improved or even achieved normal function. However, the prognosis of such blood vessels is still unclear and needs further observation.

## Conclusion

We observed an overall regression of the NVE area following PRP treatment in patients with PDR, which started 1 week after the first session and lasted for at least 3 months. OCTA provided quantitative information on vascular changes and may play an important role in monitoring the efficacy of treatment regimens in these patients.

## Data Availability

The authors had full access to all the data in the study and take responsibility for the integrity of the data and the accuracy of the data analysis as well as the decision to submit for publication.

## Abbreviations

DR: Diabetic retinopathy
PDR: Proliferative diabetic retinopathy
NVD: Retinal neovascularization at the disc
NVE: Retinal neovascularization elsewhere
PRP: Panretinal photocoagulation
NV: Neovascularization
OCTA: Optical coherence tomography angiography
FFA: Fundus fluorescein angiography
DME: Diabetic macular oedema
BCVA: Best-corrected visual acuity
GEE: Generalized estimating equations
ICC: Intraclass correlation coefficients
EFI: Extended field imaging
